# Impact of the COVID-19 Pandemic on Thyroid Cancer Surgery

**DOI:** 10.3390/curroncol31060263

**Published:** 2024-06-19

**Authors:** Max L. Lee, Uchechukwu C. Megwalu, Andrey Finegersh, Julia E. Noel, Michelle M. Chen

**Affiliations:** 1 Stanford University School of Medicine, Stanford, CA 94305, USA; maxlee12@stanford.edu; 2 Department of Otolaryngology--Head & Neck Surgery, Stanford University, Palo Alto, CA 94304, USA; umegwalu@stanford.edu (U.C.M.); afinegersh@stanford.edu (A.F.); jnoel@stanford.edu (J.E.N.); 3 Santa Clara Valley Medical Center, San Jose, CA 95128, USA; 4 ValleyCare Pleasanton Cancer Center, Pleasanton, CA 94588, USA

**Keywords:** thyroid cancer, COVID-19, pandemic, treatment delays, disparities, otolaryngology, oncology

## Abstract

The COVID-19 pandemic caused major disruptions to healthcare services in 2020, delaying cancer diagnosis and treatment. While early-stage thyroid cancer often progresses slowly, it is crucial to determine whether treatment delays associated with the pandemic have impacted the clinical presentation and management of advanced-stage thyroid cancer. The purpose of our study was to determine the impact of the early COVID-19 pandemic on thyroid cancer presentation and treatment times. Utilizing the National Cancer Database, chi-squared tests and regression analyses were performed to compare patient demographic and clinical characteristics over time for 56,011 patients diagnosed with primary thyroid cancer who were treated at the Commission on Cancer-accredited sites in 2019 and 2020. We found that thyroid cancer diagnoses decreased between 2019 and 2020, with the biggest drop among patients with cT1 disease relative to other T stages. We also found that patients diagnosed with thyroid cancer in 2020 had similar treatment times to patients diagnosed in 2019, as measured by both the time between diagnosis and start of treatment and the time between surgery and start of radioactive iodine therapy. Overall, our study suggests that resources during the pandemic were allocated to patients with advanced thyroid disease, despite a decrease in diagnoses.

## 1. Introduction

The COVID-19 pandemic led to unprecedented disruptions in healthcare services including screening, diagnosis, and treatment [1,2,3]. Cancer patients were especially vulnerable to the effects of the COVID-19 pandemic, given the importance of prompt diagnosis and treatment for many types of cancer [4], as well as the increased risk of COVID-19-related complications and mortality for patients with cancer [5]. The delays in cancer treatment and diagnosis caused by the COVID-19 pandemic have led some to predict an increase in patients presenting with late-stage disease and a subsequent increase in cancer mortality in the coming years [6,7].

Studies on thyroid cancer in particular found a decrease in fine needle aspiration biopsies and thyroid cancer diagnoses in 2020 relative to 2019 [8,9]. Additionally, small single-center studies from around the world found that thyroid cancer patients during the pandemic presented with more aggressive cancers when compared to thyroid cancer patients pre-pandemic. Specifically, these patients were more likely to have extrathyroidal extension, lymph node metastases, and multiple lesions [10,11,12]. Medas et al. conducted a multi-institutional study that included 87,467 thyroid cancer patients from 157 centers, only three of which were in the United States, and found an increased occurrence of lymph node metastases and tumor size during the pandemic. Research suggests that while the number of endocrine surgeries overall decreased in 2020 relative to 2019, surgeries for thyroid cancer patients were prioritized and mostly maintained [13,14]. The reduction in surgeries for thyroid nodules was more drastic during the early stages of the pandemic as opposed to the later stages of the pandemic in mid to late 2021 [15]. One study of a university hospital in South Korea found an increase in the number of days between initial visits and surgery during the COVID-19 pandemic relative to pre-pandemic, but this delay did not meet the threshold of statistical significance [16].

Thyroid cancer is relatively unique in that there has been a recent rise in incidence largely attributed to overdiagnosis from screening, as well as from biopsies of nodules incidentally discovered on routine imaging, though some studies suggest that this trend has flattened or even reversed within the past decade [17,18,19,20]. Even prior to the pandemic, there was a shift in American Thyroid Association (ATA) guidelines aimed at minimizing the overdiagnosis of small, incidental thyroid cancers that may not be clinically significant [18,21]. Additionally, the evidence is mixed on whether a modest delay of radioactive iodine (RAI) treatment for thyroid cancer negatively affects outcomes. While many papers suggest that modestly delayed RAI made no difference in overall survival or disease-free survival [16,22,23,24], others found that modestly delayed RAI was associated with worse overall survival and incomplete response to therapy [25,26].

Although early-stage thyroid cancer typically follows an indolent course, it is crucial to understand if delays have affected the presentation and treatment of thyroid malignancies. Given that both patients and healthcare facilities postponed less urgent healthcare services during the early COVID-19 pandemic, patients with thyroid cancer may have been at higher risk of delaying essential treatment compared to those with other types of cancer [27]. The goal of our study was to determine how the early stages of the COVID-19 pandemic impacted treatment times and the stage at presentation of thyroid cancer patients in the United States.

## 2. Materials and Methods

### 2.1. Data Source

The data source used for this study was the National Cancer Database, a hospital-based cancer registry managed by the American Cancer Society and the American College of Surgeons Commission on Cancer. This database is collected from over 1500 facilities across the United States and captures over 70% of all patients newly diagnosed with cancer in the United States [28]. This study was approved by the Stanford University School of Medicine. This is a deidentified database and was determined to be exempt from review by the Stanford University institutional review board.

### 2.2. Study Population and Covariates

The study population included adult patients with well-differentiated thyroid cancer from 2019 to 2020 who were treated at CoC-accredited cancer sites. We included only patients with papillary or follicular histologies. Sociodemographic variables that were included as covariables included age, sex, race, education, household income, distance from treatment facility, insurance status, facility region, and rural–urban classification. Both education and household income were based on the patient’s zip code and classified into quartiles based on US Census data. Insurance status was divided into the following groups: private insurance or managed care, Medicaid, Medicare, and other/government. Facility regions were divided into Northeast, Midwest, West, and South based on the regions used by the US Census Bureau [29]. The rural–urban classification was categorized as metro, urban, rural, and unknown. Distance from the treating facility was grouped into 0–10 miles, 11–20 miles, 21–50 miles, 51–100 miles, and greater than 100 miles.

The clinical variables we analyzed included clinical and pathologic TNM staging, Charlson–Deyo Score, and readmission status. Clinical and pathologic T, N, and M staging were classified according to the Eighth Edition of the American Joint Committee on Cancer (AJCC) Staging Manual [30]. The Charlson–Deyo Score was divided into two categories: the first group included patients with a Charlson–Deyo score of 0 or 1 and the second group included patients with a Charlson–Deyo score of 2 or 3. Readmission status was categorized as planned readmission, unplanned readmission, or unknown.

Descriptive statistics showing general trends in thyroid cancer diagnoses and clinical T staging over time were calculated and reported for adult patients with well-differentiated papillary or follicular thyroid cancer treated at CoC-accredited cancer sites between 2004 and 2020. However, the univariable and multivariable analyses were restricted to patients treated between 2019 and 2020 in order to assess the impact of the COVID-19 pandemic.

### 2.3. Statistical Analysis

The average annual percentage change (AAPC) and 95% confidence intervals (CIs) were used for the calculation of descriptive calculated for thyroid cancer diagnoses and clinical characteristics from 2004 to 2019. Chi-square tests were used to compare differences in patient sociodemographic and clinical characteristics between 2019 and 2020. Multivariable linear regression was performed using sociodemographic and clinical characteristics as covariates. For regression analyses, only patients diagnosed with thyroid cancer between 2019 and 2020 were included. Our treatment time variables were the number of days between diagnosis and the start of any treatment and the number of days between surgery and the start of RAI treatment. Chi-square *p*-values < 0.05 were considered statistically significant. All analysis was performed using STATA (Version 15.1; StataCorp LLC, College Station, TX, USA). The Strengthening the Reporting of Observational Studies in Epidemiology (STROBE) guidelines were used to guide the formatting of the manuscript and reporting of the results [31].

## 3. Results

### 3.1. Changes in Thyroid Cancer Demographics and Treatment Times from 2004 to 2020

**Figure 1:**
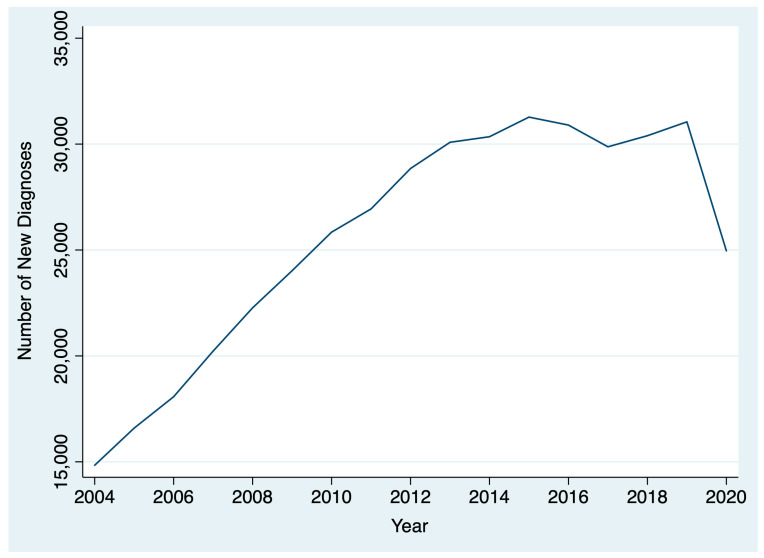
Change in thyroid cancer diagnoses over time.

**Figure 2:**
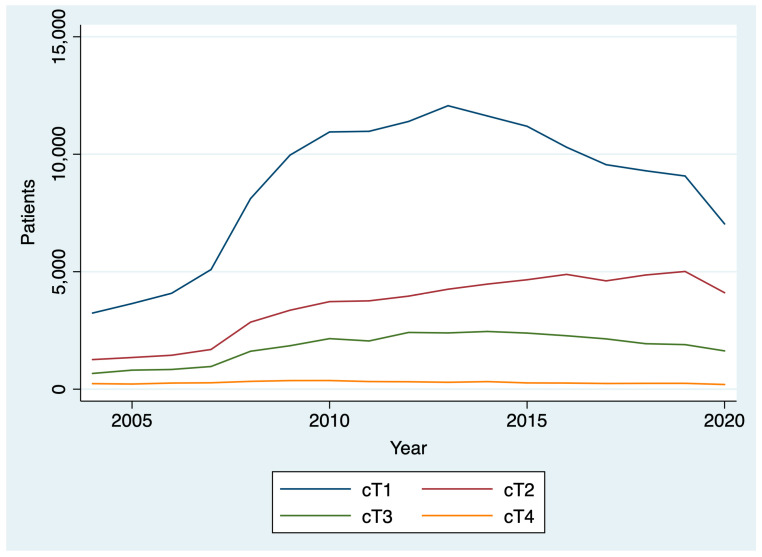
Change in clinical T stage over time.

Between 2004 and 2020, 436,546 patients were diagnosed with thyroid cancer. The AAPC in thyroid cancer diagnoses between 2004 and 2019 was 5.1% (95% CI: 2.8% to 7.5%), reflecting a general upward trend. However, between 2019 and 2020, there was a 19.6% decrease in thyroid cancer diagnoses (Figure 1). After stratifying by T stage, we found that there was a larger drop between 2019 and 2020 for patients diagnosed with cT1 disease (22.5%; chi-square *p* < 0.001) compared to patients diagnosed with cT2, cT3, and cT4 disease (18.1%, and 14.1%, 19.7%, respectively; chi-square *p* < 0.001) (Figure 2). By N stage, the drop in the number of patients diagnosed with cN0 from 2019 to 2020 was only slightly higher than the drop in the number of patients diagnosed with cN+ disease (21.4% and 20.8%, respectively; chi-square *p* = 0.005). Similarly, the drop in the number of patients diagnosed with cM0 disease from 2019 to 2020 was slightly higher than the number of patients diagnosed with cM0+ disease (21.1% and 19.1%, respectively; chi-square *p* = 0.004).

When we evaluated treatment time metrics without adjusting for clinical and demographic variables, we found that there was a gradual increase in the number of days between diagnosis and the start of any treatment for thyroid cancer between 2004 and 2019 (AAPC 3.5%, 95% CI: 2.2% to 4.8%) and a relatively flat trend in the number of days between surgery and RAI treatment for thyroid cancer (AAPC 2.4%, 95% CI: −0.8% to 4.1%). Between 2019 and 2020, there was a 6.4% drop (1.72 days) in the average number of days between diagnosis and treatment start from and a 2.4% drop (2.29 days) in the average number of days between surgery and RAI treatment.

### 3.2. Clinical and Demographic Characteristics of Thyroid Cancer Patients in 2020 versus 2019

We identified 56,011 patients who were diagnosed with thyroid cancer between 2019 and 2020. Patient demographic characteristics are shown in Table 1 and were compared to patients diagnosed in 2019 and patients diagnosed in 2020. Age, sex, race, education, and income levels were similar between the two groups. Patients diagnosed in 2020 were more likely to live further away from the treatment facility than patients diagnosed in 2019, with a higher proportion of patients living more than 100 miles away from the treatment facility (20.0% vs. 18.7%; chi-square *p* = 0.002). Patients diagnosed in 2020, compared to patients diagnosed in 2019, were less likely to be uninsured (3.5% vs. 3.9%) and more likely to be insured by Medicaid (20.1% vs. 9.6%) or Medicare (23.5% vs. 22.9%; chi-square *p* = 0.002 for all insurance status comparisons). 

In terms of clinical staging at presentation, patients diagnosed in 2020 were less likely to present with cT1 disease than those diagnosed in 2019 (40.9% vs. 41.3%, chi-square *p* < 0.001). Patients diagnosed in 2020 were also less likely to present with cN0 cancer (53.4% vs. 54.6%; chi-square *p* = 0.005) and cM0 cancer (67.9% vs. 69.2%; chi-square *p* = 0.004) (Table 2).

### 3.3. Linear Regression Analysis

After adjusting for clinical and demographic characteristics, patients diagnosed with thyroid cancer in 2020 had a shorter interval between diagnosis and the start of treatment than those diagnosed in 2019 (mean difference = −1.46 days; 95% CI, −2.52 to −0.40 (Table 3). Other covariates that had a statistically significant relationship included age, sex, distance from treatment facility, clinical T staging, and clinical M staging. Patients who were 71 years or older had longer treatment times than younger patients (mean difference = 4.35 days; 95% CI, 1.69 to 7.01). Patients who lived between 51–100 miles (mean difference = 5.05 days; 95% CI, 2.77 to 7.33) and 100 miles or greater (mean difference = 10.02 days; 95% CI, 6.89 to 13.16) from the treating facility where much more likely to have delays than those who lived closer. Compared to cT1 cancer, cT2 (mean difference = −6.34 days; 95% CI, −7.79 to −4.90), cT3 (mean difference = −16.93 days; 95% CI, −18.74 to −15.13), and cT4 (mean difference = −10.64 days; 95% CI, −15.85 to −5.43), cancers had a shorter time from diagnosis to treatment.

We performed a similar analysis to determine whether the COVID-19 pandemic resulted in any change in the number of days between surgery and the start of RAI. After adjusting for covariates, there was no difference in time between surgery and the start of RAI for patients diagnosed with thyroid cancer in 2019 compared with 2020 (mean difference = −0.01 days; 95% CI, −0.02 to 0.01) (Table 4).

## 4. Discussion

This analysis found a gradual rise over time in thyroid cancer diagnoses and treatment times from 2004 to 2019 that reversed during the COVID-19 pandemic among patients treated at CoC-accredited cancer sites. During the COVID-19 pandemic, there was a 20% drop in the number of thyroid cancer diagnoses and a 4% decrease in the number of days between diagnosis and surgery. Among all T stages, the decrease was greatest among cT1 patients. Patients diagnosed in 2020 had a shorter interval between diagnosis and the start of treatment than those diagnosed in 2019. However, the difference was small and not clinically significant. There was no difference in the interval between surgery and the start of radioactive iodine in 2020 versus 2019.

The largest decrease in thyroid cancer diagnoses was among those with cT1 thyroid disease, though substantial decreases in those with more advanced disease were also noted. This may be driven by a decrease in the availability of fine needle aspiration biopsies (FNABs) for thyroid nodules, as well as decreased imaging intensity, leading to fewer incidental findings and delayed diagnoses. A study of hospital emergency departments in the United States noted a 43% decrease in visits and a 12% reduction in imaging throughout the pandemic [32]. Small-scale studies of thyroid cancer patients in other countries have reported a 64% decrease in weekly FNABs in May through July 2020 compared to 2019 and early 2020, resulting in a 12% decrease in benign diagnoses and a 6% increase in malignant diagnoses relative to pre-pandemic [9]. The ramifications of the pandemic-driven decline in thyroid cancer diagnoses will have to be monitored closely in the coming years to determine if there is a true increase in advanced thyroid cancer cases due to diagnostic delays.

Among patients who were diagnosed with thyroid cancer in 2020, treatment following diagnosis was timelier than in 2019, although the difference was small and not clinically significant. Our study suggests that the COVID-19 pandemic did not lead to delays in the treatment of thyroid cancer. In other countries, most studies have demonstrated a stable number of thyroid cancer surgeries throughout the pandemic, despite a decline in endocrine surgeries overall, suggesting priority for oncologic care. One study from Turkey found that the annual rate of parathyroidectomy and thyroidectomy for benign goiter decreased in 2020 relative to 2019, but it did not find significant differences in thyroid cancer surgery rates between the two years [13]. Similarly, a nationwide study in Italy found a decrease in the overall number of endocrine surgeries, but with surgeries for oncological patients being maintained [14]. However, a national study in Brazil found that FNABs, oncologic thyroidectomies, and RAI treatments decreased in 2020 relative to 2019, with most procedures, excluding RAI, rebounding closer to pre-pandemic volumes in 2021 [33]. One potential explanation for these conflicting findings is variation in institution-specific pandemic response, as well as differing degrees of the burden of the COVID-19 outbreak on hospital resources and staffing. Research on breast cancer surgery found that, on average, mandated operating room shutdowns during the COVID-19 pandemic in 2020 did not lead to significant treatment delays in a subset of New York City public hospitals. However, there was wide variation between centers, with some being well-equipped to prioritize cancer surgeries and maintain treatment efficiency, while others were less successful and experienced long treatment delays [34].

Patients who benefited from timely thyroid cancer treatment during the pandemic were disproportionately younger, female, and lived closer to the treating facility. A qualitative study of older adults diagnosed with cancer demonstrated that some of the disruptions to cancer care for these patients during the pandemic included physical and social isolation, fear of contracting the virus, and difficulty with telehealth use [35]. Research on thyroid cancer patients in the pre-pandemic era found no significant delay in days to surgery by patient sex [36]. Studies suggest that because thyroid cancer is more commonly diagnosed among women, it is likely that male patients are at a higher risk of being diagnosed later compared to female patients, making timely treatment important [37]. Additionally, even prior to the pandemic, longer distances to the treating facility have been associated with delays in thyroid surgery, as well as presentation with more advanced disease [36]. No studies to date have examined whether the pandemic has exacerbated distance-related disparities in the context of thyroid cancer treatment, making it an interesting point for future research. Further work should focus on how to provide technological support, outreach, and care to our most vulnerable patients during similar situations in the future.

Limitations to our study include the lack of clinical data beyond 2020, preventing a comprehensive assessment of longer-term clinical outcomes post-pandemic. We also did not have data on thyroid ultrasounds or FNABs during the pandemic. Additionally, since the National Cancer Database (NCDB) is a hospital-based registry, our study does not include data from facilities that are not Commission on Cancer (CoC) accredited, limiting the generalizability of our findings and our ability to calculate incidence rates. The absence of data on disease-specific survival also constrains our ability to fully understand the impact of the pandemic on patient outcomes.

The COVID-19 pandemic led to a systemic decrease in the number of thyroid cancer cases diagnosed in the United States among both patients with early and advanced-stage disease, though the greatest decrease was found in early-stage disease. Older patients and patients who lived further away from the treating facility were more likely to experience treatment delays. However, our study suggests that, overall, hospitals were able to provide timely thyroid cancer treatment and effectively prioritize advanced-stage disease. Further work is necessary to determine the long-term impact of the decline in thyroid cancer diagnoses and surgeries performed.

## Figures and Tables

**Table 1:** Patient demographic and clinical characteristics, 2019 versus 2020.

	2019 N (%)	2020 N (%)	*p*-Value
**Age (in years)**			
≤50	14,962 (48.19)	12,052 (48.28)	0.265
Between 51 and 60	6624 (21.33)	5174 (20.73)	
Between 61 and 70	5745 (18.50)	4660 (18.67)	
Older than 70	3719 (11.98)	3075 (12.32)	
**Sex**			
Male	8134 (26.20)	6659 (26.68)	0.199
Female	22,916 (73.80)	18,302 (73.32)	
**Race**			
White	25,080 (80.77)	20,210 (80.97)	0.221
Black	2427 (7.82)	2006 (8.04)	
Other	3543 (11.41)	2745 (11.00)	
**Proportion of adults from patient’s zip code not graduating high school, 2000 US Census data**			
29.0%	4061 (15.88)	3238 (15.97)	0.942
20% to 28.9%	5448 (21.31)	4358 (21.49)	
14% to 19.9%	5966 (23.33)	4701 (23.19)	
Less than 14%	10,094 (39.48)	7978 (39.35)	
**Median household income for patient’s zip code, 2000 US Census data**			0.081
<USD30,000	2715 (10.62)	2252 (11.10)	
USD30,000–USD34,999	3910 (15.29)	3130 (15.43)	
USD35,000–USD45,999	6768 (26.47)	5469 (26.96)	
USD46,000+	12,179 (47.63)	9431 (46.50)	
**Distance from Facility (miles)**			0.002
≤10	11,900 (38.49)	9325 (37.52)	
11 to 20	6055 (19.58)	4771 (19.20)	
21 to 50	5261 (17.01)	4195 (16.88)	
51 to 100	1912 (6.18)	1583 (6.37)	
>100	5793 (18.73)	4977 (20.03)	
**Insurance Status**			
Uninsured	1200 (3.86)	860 (3.45)	=0.002
Private Insurance/Managed Care	19,276 (62.08)	15,281 (61.22)	
Medicaid	2980 (9.60)	2524 (10.11)	
Medicare	7108 (22.89)	5853 (23.45)	
Other	486 (1.57)	443 (1.77)	
**Charlson–Deyo Score**			
Score of 0 or 1	29,317 (94.42)	23,506 (94.17)	0.208
Score of 2 or 3	1733 (5.58)	1455 (5.83)	
**Readmission**			
No unplanned readmission	30,359 (97.77)	24,359 (97.59)	0.288
Unplanned readmission	429 (1.38)	364 (1.46)	
Unknown	262 (0.84)	238 (0.95)	
**Margins**			
Negative	25,846 (83.24)	20,753 (83.14)	0.366
Positive	3241 (10.44)	2562 (10.26)	
Unknown	1963 (6.32)	1646 (6.59)	
**Region**			0.001
East	5927 (19.09)	4235 (16.97)	
South	5141 (16.56)	4288 (17.18)	
Midwest	7735 (24.91)	6409 (25.68)	
West	4145 (13.35)	3257 (13.05)	
Unknown	8102 (26.09)	6772 (27.13)	
**Rural–Urban**			0.003
Metro	26,132 (84.16)	20,932 (83.86)	
Urban	3690 (11.88)	3094 (12.40)	
Rural	449 (1.45)	403 (1.61)	
Unknown	779 (2.51)	532 (2.13)	

**Table 2:** Patient pathological and clinical staging statistics, 2019 versus 2020.

	2019 (N%)	2020 N(%)	*p*-Value
**Pathological TNM Stage**			0.030
Stage 1	22,788 (73.39)	18,495 (74.10)	
Stage 2	3463 (11.15)	2787 (11.17)	
Stage 3	303 (.98)	236 (.95)	
Stage 4	259 (.83)	241 (.97)	
Other or Unknown	4237 (13.65)	3202 (12.83)	
**Clinical T Stage**			
T1	9073 (41.26)	7034 (40.93)	0.001
T2	5011 (22.79)	4106 (23.89)	
T3	1897 (8.63)	1630 (9.48)	
T4	249 (1.13)	200 (1.16)	
Other or Unknown	5760 (26.19)	4217 (24.54)	
**Clinical N Stage**			
N0	16,955 (54.61)	13,332 (53.41)	0.005
N+	2751 (8.86)	2179 (8.73)	
Other or Unknown	11,344 (36.53)	9450 (37.86)	
**Clinical Staging M**			
M0	21,484 (69.19)	16,946 (67.89)	0.004
M1	371 (1.19)	300 (1.20)	
Other or Unknown	9195 (29.61)	7715 (30.91)	

**Table 3:** Linear regression results, time from diagnosis to start of any treatment.

Independent Variable	Mean Difference (Days)	CI	*p*-Value
**Year 2020 (ref: Year 2019)**	−1.46	−2.52 to 0.40	0.007
**Age, y (ref: ≤50 y)**			
51–60 y	1.19	−0.40 to 2.78	0.143
61–70 y	0.72	−1.14 to 2.59	0.447
71 y or older	4.35	1.69 to 7.01	0.001
**Sex (ref: Male)**	−2.60	−3.82 to −1.38	<0.001
**Race (ref: White)**			
Black	−0.88	−3.12 to 1.35	0.438
Other	7.78	5.92 to 9.64	<0.001
**Proportion of adults from patient’s zip code not graduating high school (ref: 29.0%+)**			
20% to 28.9%	−2.45	−4.44 to 0.46	0.016
14% to 19.9%	−2.07	−4.15 to 0.01	0.051
Less than 14%	−2.14	−4.33 to 0.05	0.055
**Median household income for patient’s zip code (ref: <$30,000)**			
USD30,000–USD34,999	−0.73	−3.01 to 1.55	0.531
USD35,000–USD45,999	0.69	−1.63 to 3.01	0.559
USD46,000+	0.85	−1.65 to 3.35	0.507
**Distance from treatment facility (ref: 0–10 miles away from treatment facility)**			
11–20 miles	−0.16	−1.54 to 1.22	0.818
21–50 miles	0.27	−1.20 to 1.74	0.714
51–100 miles	5.05	2.77 to 7.33	<0.001
>100 miles	10.02	6.89 to 13.16	<0.001
**Insurance Status (ref: Uninsured or Unknown)**			
Private Insurance or Managed Care	−7.93	−11.09 to −4.78	<0.001
Medicaid	−1.36	−5.05 to 2.34	0.472
Medicare	−5.92	−9.48 to −2.36	0.001
Other Government	−2.96	−8.08 to 2.17	0.258
**Charlson–Deyo Score of 2 or 3 (ref: score of 0 or 1)**	0.78	−2.04 to 3.60	0.590
**Readmission (ref: no unplanned readmission)**			
Unplanned Readmission	−0.13	−4.38 to 4.12	0.953
Unknown	0.68	−5.21 to 6.58	0.820
**Region (ref: East)**			
South	−3.93	−5.74 to −2.12	<0.001
Midwest	−1.44	−3.19 to 0.32	0.109
West	4.81	2.74 to 6.99	<0.001
Unknown	1.66	−0.18 to 3.50	0.077
**Rural/Urban (ref: Metro)**			
Urban	−6.51	−8.35 to 4.68	<0.001
Rural	−10.58	14.03 to 7.12	<0.001
Not Available/Unknown	−1.59	−5.39 to 2.21	0.412
**Clinical T Stage (ref: cT1)**			
cT2	−6.34	−7.79 to 4.90	<0.001
cT3	−16.93	−18.74 to −12.13	<0.001
cT4	−10.64	−15.85 to −5.43	<0.001
Other or Unknown	−25.56	−26.95 to −24.17	<0.001
**Clinical N Stage (ref: N0)**			
N+	0.60	1.11 to 2.30	0.491
Other/Unknown	−0.25	−2.32 to 1.82	0.814
**Clinical M Stage (ref: M0)**			
M1	10.95	4.72 to 17.18	0.001
Other/Unknown	−5.29	−8.09 to −2.49	<0.001

CI: 95% confidence interval

**Table 4:** Linear regression results, time from surgery to start of radioactive iodine.

Independent Variable	Mean Difference (Days)	CI	*p*-Value
**Year 2020 (ref: Year 2019)**	−0.01	−0.02 to 0.01	0.358
**Age, y (ref: ≤50 y)**			
51–60 y	−0.01	−0.03 to 0.01	0.542
61–70 y	−0.01	−0.03 to 0.01	0.480
71 y or older	−0.02	−0.05 to 0.01	0.193
**Sex (ref: Male)**	0.01	0.00 to 0.02	0.059
**Race (ref: White)**			
Black	−0.01	−0.04 to 0.02	0.484
Other	0.01	−0.01 to 0.03	0.224
**Proportion of adults from patient’s zip code not graduating high school (ref: 29.0%+)**			
20% to 28.9%	−0.01	−0.03 to 0.01	0.326
14% to 19.9%	−0.02	−0.05 to 0.00	0.065
Less than 14%	−0.02	−0.04 to 0.01	0.171
**Median household income for patient’s zip code (ref: <$30,000)**			
USD30,000–USD34,999	0.01	−0.01 to 0.04	0.348
USD35,000–USD45,999	0.03	0.00 to 0.05	0.056
USD46,000+	0.02	−0.01 to 0.05	0.187
**Distance from treatment facility (ref: 0–10 miles away from treatment facility)**			
11–20 miles	−0.02	−0.03 to 0.00	0.062
21–50 miles	0.01	−0.01 to 0.03	0.219
51–100 miles	0.05	0.02 to 0.08	0.001
>100 miles	0.04	0.00 to 0.07	0.049
**Insurance Status (ref: Uninsured/Unknown)**			
Private Insurance or Managed Care	0.00	−0.03 to 0.03	0.988
Medicaid	0.02	−0.02 to 0.06	0.260
Medicare	0.01	−0.03 to 0.04	0.705
Other Government	0.00	−0.06 to 0.06	0.974
**Charlson–Deyo Score of 2 or 3 (ref: score of 0 or 1)**	0.03	0.00 to 0.06	0.062
**Readmission (ref no unplanned readmission)**			
Unplanned Readmission	0.05	0.01 to 0.09	0.019
Unknown	0.02	−0.02 to 0.07	0.325
**Region (ref: East)**			
South	−0.15	−0.17 to −0.13	<0.001
Midwest	−0.10	−0.12 to −0.08	<0.001
West	−0.05	−0.07 to −0.03	<0.001
Unknown	−0.09	−0.11 to −0.07	<0.001
**Rural/Urban (ref: Metro)**			
Urban	−0.03	−0.05 to −0.01	0.015
Rural	−0.01	−0.07 to 0.04	0.595
Not Available/Unknown	−0.07	−0.12 to −0.02	0.008
**Pathologic T Stage (ref: pT 1)**			
pT2	0.02	0.00 to 0.03	0.047
pT3	0.01	−0.01 to 0.02	0.265
pT4	−0.01	−0.05 to 0.02	0.437
Other or Unknown	0.06	0.02 to 0.10	0.003
**Pathologic N Stage (ref: N0)**			
N+	0.02	0.00 to 0.03	0.041
Other/Unknown	−0.01	−0.03 to 0.01	0.190
**Pathologic M Stage (ref: M0)**			
M1	−0.02	−0.07 to 0.03	0.435
Other/Unknown	0.03	−0.01 to 0.06	0.160

CI: 95% confidence interval

## Data Availability

The data presented in this study are available from NCDB and provided by the American College of Surgeons, by application.

## Abbreviations

NCDB: National Cancer Database, CoC: Commission on Cancer, APC: Annual Percentage Change, CI: Confidence Interval, AJCC: American Joint Committee on Cancer, RAI: Radioactive Iodine, ATA: American Thyroid Association.
